# Molecular Hydrogen Reduces LPS-Induced Neuroinflammation and Promotes Recovery from Sickness Behaviour in Mice

**DOI:** 10.1371/journal.pone.0042078

**Published:** 2012-07-31

**Authors:** Stefan Spulber, Karin Edoff, Lie Hong, Shinkatsu Morisawa, Sanetaka Shirahata, Sandra Ceccatelli

**Affiliations:** 1 Department of Neuroscience, Karolinska Institutet, Stockholm, Sweden; 2 Nihon Trim Co Ltd., Osaka, Japan; 3 Department of Bioscience and Biotechnology, Faculty of Agriculture, Kyushu University, Kyushu, Japan; National Institutes of Health, United States of America

## Abstract

Molecular hydrogen has been shown to have neuroprotective effects in mouse models of acute neurodegeneration. The effect was suggested to be mediated by its free-radical scavenger properties. However, it has been shown recently that molecular hydrogen alters gene expression and protein phosphorylation. The aim of this study was to test whether chronic *ad libitum* consumption of molecular hydrogen-enriched electrochemically reduced water (H-ERW) improves the outcome of lipopolysaccharide (LPS)-induced neuroinflammation. Seven days after the initiation of H-ERW treatment, C57Bl/6 mice received a single injection of LPS (0.33 mg/kg i.p.) or an equivalent volume of vehicle. The LPS-induced sickness behaviour was assessed 2 h after the injection, and recovery was assessed by monitoring the spontaneous locomotor activity in the homecage for 72 h after the administration of LPS. The mice were killed in the acute or recovery phase, and the expression of pro- and antiinflammatory cytokines in the hippocampus was assessed by real-time PCR. We found that molecular hydrogen reduces the LPS-induced sickness behaviour and promotes recovery. These effects are associated with a shift towards anti-inflammatory gene expression profile at baseline (downregulation of TNF- α and upregulation of IL-10). In addition, molecular hydrogen increases the amplitude, but shortens the duration and promotes the extinction of neuroinflammation. Consistently, molecular hydrogen modulates the activation and gene expression in a similar fashion in immortalized murine microglia (BV-2 cell line), suggesting that the effects observed *in vivo* may involve the modulation of microglial activation. Taken together, our data point to the regulation of cytokine expression being an additional critical mechanism underlying the beneficial effects of molecular hydrogen.

## Introduction

Molecular hydrogen has been proposed as an effective antioxidant treatment [1], based on its free-radical scavenger properties, and has been successfully used in a wide range of pathological conditions involving acute oxidative stress (reviewed in [2] and [3]). The primary molecular target of molecular hydrogen is not clearly understood yet. The main mechanism of action was suggested to be the preferential scavenging of hydroxyl (HO**·**) and peroxynitrite (ONOO**·**) radicals, thereby reducing the oxidative damage to both membrane lipids and DNA [1]. In addition, recent reports indicate consistent effects on gene expression and protein phosphorylation [4], [5].

Molecular hydrogen can be delivered exogenously, *via* inhaled air or dissolved in water (dietary consumption, systemic administration, or local application), or produced by intestinal bacteria [6] (reviewed in [2]). The acute administration of hydrogen either in breathing air, or dissolved in sterile saline have been successful in improving the outcome of conditions in which reactive oxygen species (ROS) are known to play a critical role, such as ischemia-reperfusion lesions in different organs, including liver, intestine, kidney, and nervous system (reviewed in [3]). The protective effects have been associated with reduction of oxidative stress and cytokine production measured 24 h after reperfusion[7]–[11]. In addition, molecular hydrogen administered in drinking water could protect mesencephalic dopaminergic neurons from MPTP-induced degeneration in mice [12].

The nervous system responds to most injuries with neuroinflammation, which is characterized by phenotypical changes in microglia and astrocytes, and increased production of free radicals, cytokines, and neurotrophins. ROS released by activated microglia contribute to the elimination of pathogens, but also mediate the detrimental effects of neuroinflammation ([13], reviewed in [14]). In addition, ROS act as secondary messengers to activate the expression of proinflammatory cytokines [15]. A transient or chronic imbalance (as in ageing) between pro- and anti-inflammatory cytokines in the brain is associated with behavioural alterations ranging from decreased activity (see [16], [17]) to depression-like symptoms and cognitive deficits [18]. Neurotrophins, such as brain-derived neurotrophic factor (BDNF) are required for the maintenance and recovery of neuronal function. The expression of neurotrophins and their receptors has been shown to be modulated by the balance between pro- and anti-inflammatory cytokines [19]. Consistently, therapeutic strategies to prevent or reduce inflammation, including administration of antioxidants [20], [21] and non-steroid anti-inflammatory drugs (NSAID) [22], have proven successful in limiting the behavioural alterations, as well as in preventing neurodegeneration (see also [23]). The stereotypical adaptive response occurring during neuroinflammation limits the damage and promotes functional recovery. However, uncontrolled continuation of neuroinflammation may lead to neurodegeneration [24]. Therefore, the regulation of both amplitude and duration of neuroinflammation is critical for optimizing its efficiency as defense mechanism.

A commonly used model to induce neuroinflammation is via systemic administration of lipopolysaccharide (LPS). LPS is a highly conserved component of the wall of Gram-negative bacteria that is recognized by the mammalian immune system as pathogen-associated molecular pattern (PAMP). It binds to the toll-like receptor 4 (TLR4) via the adapter protein CD14 [25] and upregulates the expression of proinflammatory cytokines following the nuclear translocation of NF-kB [26]. LPS administered intracerebrally or systemically triggers the activation of microglia and the subsequent increased expression of cytokines ([27]; reviewed by [28]). Systemic administration of LPS induces, in a dose-dependent manner, transient fever, anorexia, and a global decrease in exploratory activity, social and sexual interactions [21]. The behavioural alterations are collectively referred to as “sickness behaviour” (reviewed in [29]).

Proinflammatory cytokines, such as interleukin (IL)-1 and tumour necrosis factor (TNF- α), are driving the sickness behaviour [29], while antiinflammatory cytokines, such as IL-10, have been shown to limit the sickness behaviour by regulating the LPS-induced expression of proinflammatory cytokines ([30], [31], reviewed in [17]). Proinflammatory cytokines also transiently suppress the expression of clock genes in the suprachiasmatic nucleus [32], [33], which alters the circadian rhythmicity in spontaneous locomotion and sleep patterns. The microglia are cells located in the brain parenchyma that belong to the innate immune system. They undergo rapid phenotypical changes in response to injury [34] and are a major source of proinflammatory cytokines in the brain [35]. The mounting of a neuroinflammatory response depends on the presence and the activation of microglia. Importantly, the baseline activation state of microglia is a crucial determinant of the amplitude and the duration of the sickness behaviour and cytokine production in the brain [36]. Recent evidence indicates that the microglial redox status regulation plays a central role in modulating the neuroinflammatory response ([13], [37], [38], reviewed in [39]).

In this study we have investigated the effects of chronic *ad libitum* consumption of molecular hydrogen- enriched electrochemically reduced water (H-ERW) on neuroinflammation induced by systemic administration of LPS. We found that molecular hydrogen limits the development and facilitates the resolution of sickness behaviour. In addition, it enhances the induction, and promotes the resolution of neuroinflammation, thereby presumably promoting the efficiency of the defense response. To investigate the direct effects of molecular hydrogen on isolated microglia we used a murine microglia cell line (BV-2). In agreement with the findings *in vivo*, we found that BV-2 cells cultured in H-ERW-based medium displayed a more robust response when exposed to LPS.

## Results

### Molecular Hydrogen-enriched H-ERW Reduces the Sickness Behaviour Induced by LPS

*Ad libitum* consumption of H-ERW did not have any effects on behaviour at baseline or in either sham group. To investigate whether molecular hydrogen affects the induction of sickness behaviour and/or the recovery, we assessed (1) the effect on the suppression of locomotor activity 2 h after LPS administration (acute phase), and (2) the alterations in circadian activity in the homecage during 72 h following the LPS challenge.

**Figure 1:**
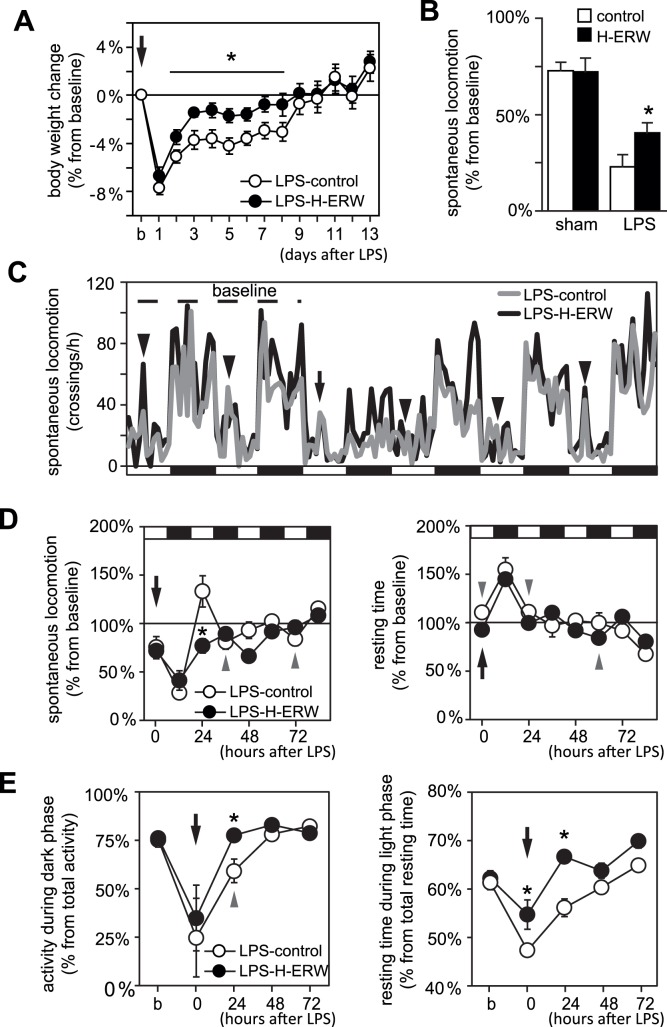
Effects of molecular hydrogen in drinking water on sickness behaviour induced by systemic administration of LPS. (A) Changes in bodyweight monitored for 2 weeks after the administration of LPS. Baseline bodyweight is calculated as average of the last 3 days preceding the administration of LPS. Mice drinking molecular hydrogen-enriched H-ERW drop slightly less than control mice, and recover faster to baseline bodyweight (N = 8 mice per group; * p<0.05, repeated measures ANOVA, followed by contrast analysis). (B) Spontaneous locomotor activity in a novel environment. The baseline activity was recorded 22 h prior to LPS and was used as reference for activity recorded 2 h after the injection of either vehicle (sham), or LPS. Note that the habituation to the testing conditions accounts for 25% decrease in locomotor activity. LPS administration decreases the locomotor activity to 25% in control mice, and to 45% in H-ERW mice (sham-control: N = 15; sham-H-ERW: N = 15; LPS-controls: N = 13; LPS-H-ERW: N = 14; * p<0.05, factorial ANOVA followed by contrast analysis). (C) Spontaneous locomotion in the homecage expressed as average number of visits per hour (see text for details) for 6 consecutive light-dark cycles (light and dark phases indicated at the bottom of the graph). The injection of LPS is marked by arrow. Arrowheads indicate the peaks in locomotor activity induced by handling for measuring bodyweight. Note that the pattern with 2 or 3 distinct peaks of activity is preserved in H-ERW mice even during the first dark phase following the LPS injection (although at lower amplitude than at baseline), and is restored earlier than in control mice. “baseline” indicates the two light-dark cycles that were used for deriving baseline values for all parameters analysed. (D) Spontaneous locomotor activity resting time in the homecage (light and dark phases are indicated at the top of the graph). Systemic administration of LPS (arrow) induces a significant decrease in spontaneous locomotion during both light and dark phases of the following circadian cycle, followed by a transient hyperactive period in control, but not in H-ERW mice. Resting time is significantly increased only during the dark phase following the LPS injection in H-ERW mice, compared to 3 consecutive phases in the control mice. Arrowheads indicate the timepoints where a significant change in spontaneous locomotion or resting time occurs in only one group (*i.e.* either LPS-control, or LPS-H-ERW). (E) Circadian distribution of locomotor activity and resting time. The circadian distribution of both activity and resting time was restored earlier in mice drinking H-ERW (* p<0.05, repeated measures ANOVA followed by contrast analysis). C, D, E–N = 8 mice per group, recorded in parallel in groups of 4 LPS-control and 4 LPS-H-ERW mice.

Systemic administration of LPS induced a decrease in bodyweight by 6–8% within the first 24 h (to a similar extent in LPS-H-ERW mice as in LPS-controls; N = 8/group) (Fig. 1), followed by a gradual recovery to baseline. The LPS-H-ERW mice recovered faster and reached baseline bodyweight earlier than LPS-controls (Fig. 1A). *Ad libitum* consumption of H-ERW did not influence the bodyweight in sham mice (*i.e.* not injected with LPS) (N = 8/treatment; data not shown).

In agreement with earlier reports, habituation accounted for a decrease to 75% from baseline spontaneous locomotion in sham-treated mice (see also [21]). Systemic administration of LPS suppressed the spontaneous locomotor activity to 25% from baseline in LPS-control mice, and to 45% in LPS-H-ERW mice (factorial ANOVA followed by contrast analysis, p<0.05) (Fig. 1B).

To follow the recovery from sickness behaviour, we monitored the activity of mice in the home-cage for at least 2 light-dark cycles before the administration of LPS, and 3 light-dark cycles following the LPS challenge (Fig. 1C). We found that the global locomotor activity was significantly suppressed immediately following the LPS injection to a similar extent in LPS-control and LPS-H-ERW mice. However, the ultradian patterns of activity (*e.g.* the 3 peaks of activity during the active phase) were preserved in LPS-H-ERW mice immediately after LPS administration, although at a lower amplitude than at baseline (Fig. 1C, see also the File S1 and Figure S1 for a more detailed analysis of the variations in amplitude in relation to sickness behaviour).

The spontaneous locomotor activity dropped to 75% of baseline during the light phase immediately following the injection of LPS, and to 28.2% and 41.8% of baseline during the following dark phase in the LPS-control and LPS-H-ERW groups respectively. During the following light-dark cycles, the control mice were briefly hyperactive compared to baseline, then returned to close-to-normal spontaneous locomotor activity in the homecage. In contrast, the LPS-H-ERW mice remained slightly hypoactive and returned to close-to-normal levels of spontaneous locomotion (Fig. 1D). The analysis of resting time was significantly increased for 3 consecutive phases in LPS-control mice (one-sample t-test, p<0.05). In contrast, the LPS-H-ERW mice displayed an increase in resting time only during the dark phase following the injection of LPS (Fig. 1D). The circadian distribution of both spontaneous locomotor activity and resting time in the homecage was restored to baseline values after 24 h in the LPS-H-ERW mice, compared to 48 h in LPS-control mice (Fig. 1E).

### Molecular Hydrogen-enriched H-ERW Induces an Antiinflammatory Baseline Cytokine Expression Profile and Promotes the Resolution of Neuroinflammation

In light of the role played by cytokines in LPS-induced neuroinflammation, we investigated the expression of proinflammatory (TNF-α, IL-1β, and IL-6) and antiinflammatory (IL-10) cytokines in the hippocampus (a brain region where neuroinflammation occurs robustly in response to brain injury or systemic inflammation [40], [41]). The timepoints for measuring the changes in gene expression were selected to match the acute phase of the sickness syndrome (2–4 h after the administration of LPS; see [29]) and the recovery of spontaneous locomotor behaviour (24–48 h).

**Figure 2:**
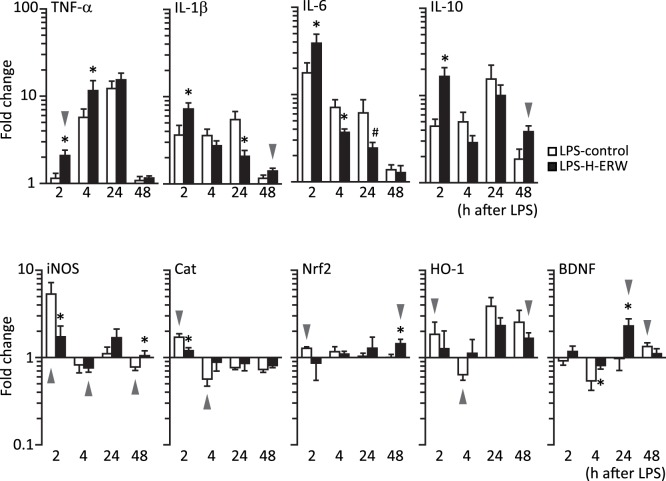
Analysis of LPS-induced changes in gene expression in the hippocampus. Data shown as fold change relative to sham mice. N = 4–5 samples/group and timepoint. Arrowheads indicate the groups and timepoints where significant gene expression regulation was found in only one of the LPS-treated groups compared to its respective sham. * - significantly different from control at the given timepoint; # - p = 0.07.

The alterations in gene expression in mouse hippocampi are shown in Table 1 and Fig. 2 (N = 4–6/group). In the hippocampus of sham-treated mice, we found that H-ERW upregulated IL-10, and downregulated TNF-α and HO-1 (one-sample hypothesis t-test, p<0.05).

**Table 1:** Regulation of gene expression in hippocampus.

gene	baseline	2 h		4 h		24 h		48 h	
	(H-ERW)	control	H-ERW	control	H-ERW	control	H-ERW	control	H-ERW
TNF-α	↓	−	↑	↑	↑	↑	↑	↑	↑
IL-1β	−	↑	↑	↑	↑	↑	↑	−	↑
IL-6	−	↑	↑	↑	↑	↑	↑	−	−
IL-10	↑	↑	↑	↑	↑	↑	↑	−	↑
iNOS	−	↑	−	−	↓	−	−	↓	−
Cat	−	↑	−	↓	−	−	−	↓	↓
Nrf2	−	↑	−	−	−	−	−	−	↑
HO1	↓	↑	−	↓	−	↑	↑	−	↑
BDNF	−	−	−	↓	↓	−	↑	↑	−

baseline: sham-H-ERW vs. sham-control; otherwise LPS-control or LPS-H-ERW vs. “no-regulation” hypothesis (one sample t-test versus fold-regulation = 1);

↑ - significant upregulation;

↓ - significant downregulation.

Following the administration of LPS, TNF-α was upregulated at 4, 24 h, and 48 h in the hippocampi of control mice, and at all timepoints (2, 4, 24, 48 h) in H-ERW mice. The upregulation was significantly higher in the H-ERW mice 2 and 4 h after systemic challenge with LPS.

IL-1β was upregulated in LPS-control mice at 2, 4, and 24 h, but not at 48 h. In LPS-H-ERW mice, we found IL-1β to be upregulated to a higher extent at 2 h, and to a significantly lower extent at 24 h.

IL-6 was upregulated at 2, 4, and 24 h, but not at 48 h, in both groups. In LPS-H-ERW mice, the upregulation of IL-6 was higher than in LPS-controls at 2 h, and lower than in LPS-controls at 4 and 24 h after exposure to LPS.

Similarly, IL-10 was upregulated in LPS-control mice at 2, 4, and 24 h, but not at 48 h. In LPS-H-ERW mice, IL-10 upregulation was higher at 2 h, and was still detectable 48 h after exposure to LPS.

LPS-induced neuroinflammation is accompanied by an increased production of free radicals and changes in the redox homeostasis in microglial cells [38]. This induces a transient upregulation of proinflammatory cytokines (via activation of NF-kB) and of phase II antioxidant and detoxification enzymes, such as heme oxygenase-1 (HO-1) (via activation of Nrf2, which binds to antioxidant response elements, ARE) (reviewed in [39]). Importantly, HO-1 is involved in the extinction of neuroinflammation [37]. Nitric oxide (NO) produced and released in the extracellular space by activated microglia is critical for sustaining the activation and mediates the neurotoxic effects of activated microglia [42]. The control of iNOS expression is very complex and includes NF-kB activation as well as ARE-binding transcription factors (such as Nrf2) (reviewed in [43]). The antioxidant enzyme catalase (Cat) is upregulated promptly in response to changes in the redox status and inhibits the LPS-induced activation and NO production in microglia [15]. Therefore, we assessed the changes in gene expression for iNOS, Cat, Nrf2 and HO-1, as marker of Nrf2 transcription-regulation activity.

The expression of iNOS was upregulated at 2 h, and downregulated at 48 h in the hippocampi of LPS-control mice. In LPS-H-ERW mice, iNOS was downregulated only 4 h after exposure to LPS, and was significantly different from controls at 2 and 48 h after exposure.

In the hippocampi of LPS-control mice, Cat was upregulated at 2 h, and downregulated at 4 and 48 h after exposure to LPS. In the LPS-H-ERW mice, Cat was downregulated only 48 h after exposure to LPS, and was lower than in LPS-controls at 2 h after exposure.

Nrf2 was upregulated 2 h in LPS-controls, and 48 h in LPS-H-ERW mice, at which point its expression was higher than in LPS-controls.

HO-1 was downregulated at 4 h and upregulated at 2 and 24 h after exposure to LPS in LPS-control mice, whereas in LPS-H-ERW mice it was upregulated both 24 and 48 h after exposure to LPS. However, no significant differences were found between the two LPS groups.

A peripheral immune stimulation is accompanied by a transient alteration in performance in hippocampal-dependent tasks [44], [45], as well as by a downregulation of plasticity-related genes, including BDNF [46], [47]. The functional recovery after brain injury and neuroinflammation is associated with an upregulation of BDNF expression [48], [49]. We measured the expression of BDNF in the hippocampus following systemic administration of LPS and found a significant downregulation 4 h after LPS administration in both groups, but to a lesser extent in the H-ERW mice. BDNF was robustly upregulated only in H-ERW mice at 24 h, while in control mice the upregulation was detectable only 48 h after exposure to LPS.

In summary, molecular hydrogen induces a more robust induction of proinflammatory (TNF-α, IL-1β, IL-6) and antiinflammatory (IL-10) cytokines in the acute phase (2–4 h), followed by a faster decay in expression at later timepoints (24 h). The stronger induction of proinflammatory cytokines in the H-ERW-treated mice at early timepoints was accompanied by lower expression levels of iNOS and Cat 2 h after the administration of LPS. The effect of molecular hydrogen in drinking water at the timepoints corresponding to the recovery from sickness syndrome (24–48 h) is characterized by a lower upregulation of proinflammatory cytokines (IL-1β and IL-6), an earlier upregulation of BDNF, and an upregulation of IL-10, HO-1, and Nrf2 48 h after LPS administration only in H-ERW-treated mice.

### Molecular Hydrogen-enriched Culture Medium Alters the Proliferation and Activation of BV-2 Microglial Cells Following the Exposure to LPS, but not in Baseline Conditions

Next, we investigated whether the pretreatment with hydrogen-enriched culture medium could modulate the activation of microglia induced by exposure to LPS. To this end we used BV-2 cells (a murine microglia-derived cell line), which have been shown to be a reliable in vitro model for microglial activation[50]–[54] and display up to 90% similar profile of gene expression regulation as compared to primary microglia cultures [55].

**Figure 3:**
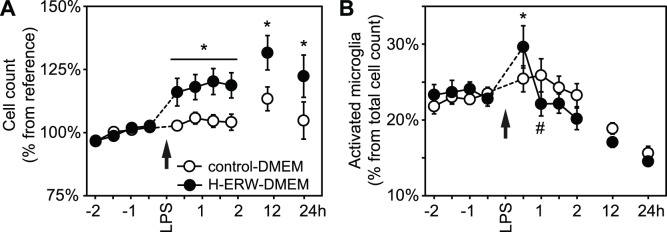
Longitudinal analysis of proliferation and activation upon exposure to LPS. BV-2 cells were grown in DMEM supplemented with 10% FCS for 24 h after plating. The growth medium was replaced with serum free control- or H-ERW-DMEM, immediately before starting the recording. LPS was added to the growth medium after 24 h, and the cells were followed for an additional 24 h. (A) Total cell number relative to the average over the last 2 h before exposure to LPS. (B) Activation of BV-2 cells by exposure to LPS 1 µg/ml assessed by automated analysis of cell morphology (% from total cell number).

First, we used the Cell-IQ2™ system for the continuous monitoring of cell counts and morphological classification. We found that BV-2 cells have an exponential growth curve for at least 24 h after removing the fetal calf serum (FCS). The curve parameters were similar in BV-2 microglia cultured in either control- or H-ERW-DMEM, and the time constant was (2.3±0.1) 10^−2^ h^−1^. This corresponds to a relative increase in cell number of approximately 2.3%/h. The automated classification based on morphological analysis also found similar proportions of activated microglia in both groups at baseline. The addition of LPS to the growth medium induced a brief but significant response only in the H-ERW-DMEM cells (Fig. 3; repeated measures ANOVA for trend control-DMEM: p = 0.20; H-ERW-DMEM: p<0.05). Thus, LPS induced a rapid change in total cell counts that was maintained for up to 24 h, and a transient increase in the proportion of activated cells (from 24.1±0.9% to 29.7±2.8%) followed by a trend towards reduced activation by 1 h after LPS.

### Molecular Hydrogen-enriched Culture Medium Alters Gene Expression in BV-2 Microglia Both at Baseline and after Exposure to LPS

**Figure 4:**
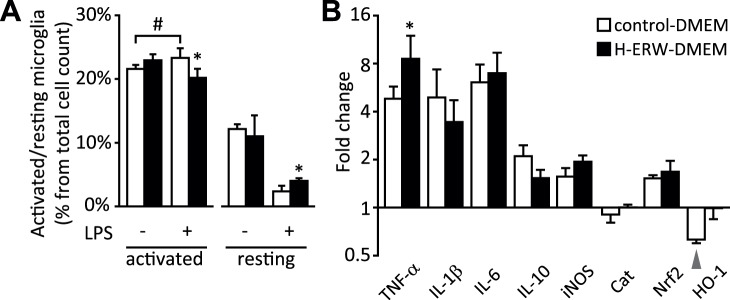
Analysis of activation and gene expression profile in BV-2 cells after 2 h of exposure to LPS. (A) Quantification of activation. The BV-2 cells cultured in H-ERW-DMEM display a smaller proportion of activated cells and a larger proportion of resting cells after exposure to LPS for 2 h. (B) Gene expression regulation after 2 h exposure to LPS. In BV-2 cells grown in H-ERW-DMEM, the upregulation of TNF-α is higher, while HO-1 is not downregulated by LPS (* - p<0.05 two-tailed t-test).

To further investigate the phenotypical changes induces by LPS, we harvested the cells 2 h after the exposure to LPS and analysed the activation state and the changes in gene expression by quantitative real time PCR. We found that cells grown in H-ERW-DMEM show less signs of activation (lower proportion of activated cells, and higher proportion of resting cells; see Fig. 4A) after 2 h of exposure to LPS, but not in normal conditions. IL-10 was significantly upregulated in BV-2 cells cultured in H-ERW-DMEM at baseline (Table 2). Following the exposure to LPS, we found TNF-α, IL-1β, IL-6, IL-10, iNOS, and Nrf2 to be upregulated in cells grown in both control- and H-ERW-DMEM, and HO-1 to be downregulated only in the cells grown in control-DMEM (Table 2). The expression of TNF-α was significantly higher in the cells grown in H-ERW-DMEM (Fig. 4B).

**Table 2:** Regulation of gene expression in BV-2 cells.

	baseline	2 h	
gene	(H-ERW)	control	H-ERW
TNF-α	−	↑	↑
IL-1β	−	−	−
IL-6	−	↑	↑
IL-10	↑	↑	↑
iNOS	−	↑	↑
Cat	−	−	−
Nrf2	−	↑	↑
HO1	−	↓	−

baseline: sham-H-ERW vs. sham-control; otherwise LPS-control or LPS-H-ERW vs. “no-regulation” hypothesis (one sample t-test versus fold-regulation = 1);

↑ - significant upregulation;

↓ - significant downregulation.

## Discussion

We found that *ad libitum* consumption of molecular hydrogen-enriched water reduces the LPS-induced sickness behaviour and promotes recovery. These effects are associated with a shift towards anti-inflammatory gene expression profile already at baseline (downregulation of TNF-α and upregulation of IL-10). In addition, the pattern of gene expression regulation following systemic administration of LPS indicates that molecular hydrogen increases the amplitude, but shortens the duration and promotes the extinction of the neuroinflammatory response. Consistently, molecular hydrogen modulates the activation and gene expression in a similar fashion in immortalized murine microglia (BV-2 cell line), suggesting that the effects observed *in vivo* may involve the modulation of microglial activation.

Previous studies using acute or chronic administration of molecular hydrogen after ischemia-reperfusion lesions in different organs have consistently described lower proinflammatory cytokine levels and oxidative stress [8], [9], [56], [57]. However, the molecular hydrogen was administered within a limited time frame after the lesion, which did not include the acute phase of the inflammatory response. To our knowledge, this is the first study to assess the effects of molecular hydrogen on the very early phases of inflammation.

We found an apparently paradoxical increase in amplitude in neuroinflammation underlying the milder behavioural alterations induced by systemic LPS administration. Previous reports indicate that a less severe sickness behaviour is associated with a decrease in NF-kB activation and pro-inflammatory cytokine production in the brain [20]. IL-10 is a potent repressor of proinflammatory cytokine expression, and genetic ablation of IL-10 expression prolongs the time required for the LPS-induced behavioural changes and downregulation of BDNF to subside [58]. In this context, it appears that the antiinflammatory cytokine expression profile at baseline, as well as the faster upregulation of TNF-α, IL-1 and IL-6, favoured a stronger upregulation of IL-10 at 2 h in the LPS-H-ERW mice, which in turn suppressed the IL-1 and IL-6 at later timepoints. Thus, the antiinflammatory cytokine expression profile outweighs the suppressive effects of proinflammatory cytokines on behavior in the acute phase.

The timecourse of gene expression regulation in the hippocampus indicates that H-ERW induces a more robust induction of the neuroinflammatory response, followed by a faster extinction of neuroinflammation and sickness behaviour. As demonstrated earlier, experimentally-induced seizures increase in severity in mice lacking functional IL-6 signalling [59], while a sustained upregulation of proinflammatory cytokines is predictive for a worse outcome [36], suggesting that proper induction, as well as timely resolution of neuroinflammation are required for a favourable behavioural outcome.

Twenty-four hours after systemic administration of LPS, most behavioural parameters (total resting time during the light phase, and the circadian distribution of spontaneous locomotor activity and resting) were already restored in molecular hydrogen-enriched H-ERW mice, but not in controls. Consistently, the behavioural parameters were associated with a lower upregulation of IL-1β and IL-6 (still significantly above the expression levels in shams), and with an upregulation of BDNF only in H-ERW-treated mice. At 48 h, the expression of IL-10, Nrf2, and HO-1 was still upregulated in H-ERW-treated mice, suggesting a stronger anti-inflammatory response aiming at restoring the redox homeostasis and functional recovery. The activation Nrf2 transcription factor and the upregulation of HO-1 are important for extinguishing the neuroinflammatory response and promoting recovery ([37], [60], reviewed in [39]). Thus, our data support the hypothesis that the effects of chronic ingestion of molecular hydrogen are mediated at least in part by an increase in gene transcription downstream of Nrf2 (see also [61], [62]).

The microglia respond very rapidly (within minutes) to brain injury with phenotypical alterations at morphological, redox, and protein expression level (see [34], [35]). Importantly, the outcome of neuroinflammation greatly depends on the baseline functional state of microglia (*e.g.* microglial priming, characteristic for the ageing brain, is typically associated with slower recovery from sickness syndrome induced by LPS, [36]). We found that molecular hydrogen in drinking water alters the regulation of gene expression in baseline conditions and therefore we investigated the effects of pre-exposure to molecular hydrogen-enriched cell culture medium (H-ERW-DMEM) on microglial activation induced by LPS. In line with the findings in mouse hippocampus, IL-10 was upregulated at baseline in BV-2 microglia cultured in H-ERW-DMEM. Following the exposure to LPS, we found evidence of activation already after 1 h. Importantly, the variation in proportion of activated cells was significant only in the cells cultured in H-ERW-DMEM, where the response appeared earlier and subsided faster than the trend identifiable in controls, in agreement with the pattern of neuroinflammation found *in vivo*. In addition to the changes in microglial morphology, suggesting lower activation after 2 h of exposure to LPS, we found a higher upregulation of TNF-α, and no downregulation of HO-1 in BV-2 cells cultured in H-ERW-DMEM. Resting microglia in culture express almost exclusively type 2 TNF-α receptors (TNFR2), and their stimulation activates the transcription of mainly antiinflammatory molecules, such as IL-10 [63]. This is in line with a recent report on molecular hydrogen inhibiting the LPS-induced NO production and the activation of proinflammatory signalling cascades (p38 MAPK, JNK and NF-kB) in RAW264 macrophage cell line [4], and in agreement with the recovery-promoting gene expression profile we found *in vivo* (see also Fig. S2).

In conclusion, we bring evidence that chronic consumption of hydrogen dissolved in drinking water improves the acute behavioural outcome and promotes the recovery from neuroinflammation induced by systemic administration of LPS. This appears to be due to a modulating effect on the innate immune response in the brain. Altogether, our data point to regulation of cytokine expression being a critical additional mechanism underlying the beneficial effect of molecular hydrogen.

## Materials and Methods

### Animals

Young adult male C57Bl/6 mice (10 week old; Charles River, Germany) were housed 4 per cage under controlled environmental conditions (20.5–22.5°C; relative humidity 60%; light:dark 12 h:12 h, lights on 6 a.m.) with free access to water and standard pelleted food. Within a week after arrival in the animal facility, the animals were tagged with subcutaneous radio frequency transponders (Trovan Ltd., UK or DataMars SA, Switzerland) under brief Isoflurane anesthesia (4% in air). The experimental procedures (*e.g.* the initiation of H-ERW administration) were intiated at least 3 days after the implantation of RFID tags (*i.e.* after the correct placement was confirmed and the insertion wounds were completely closed). All procedures were performed in agreement with European regulation and were approved by the Ethical Committee Stockholm North (ethical permit number N373/09).

### Treatments

Water enriched in dissolved molecular hydrogen (H-ERW) was produced using a Trim Ion TI-9000 water treatment machine (Nihon Trim Co. Ltd., Osaka, Japan). Tap water is first passed through an activated charcoal filter for removal of bacteria and other microscopic impurities, then subject to electrolysis for enrichment in hydrogen. Filtered water (not enriched in hydrogen) was used as control. The microbiological analysis showed that the water is virtually pathogen-free, thus suitable for both *in vivo* and *in vitro* experiments. The properties of the water were regularly tested for consistency (see Table 3).

**Table 3:** Characteristics of the water.

	Tap water	control	H-ERW	control-DMEM#	H-ERW-DMEM#
pH	8.3±0.1	7.9±0.2	10.5±0.1	7.2±0.1	7.2±0.1
ORP (mV)	144.3±14.9	113.7±21.8	−261.7±12.5	33.7±5.1	−34.2±4.2
DH (ppb, µg l^−1^)	2.4±0.4	6.2±1.2	510.8±21.9	1.8±1.0	78.5±20.1
ΔDH/Δt (µg l^−1^ h^−1^)*	N/A	N/A	−59.4±6.7	N/A	N/A

All measurements performed at room temperature (22±1°C). FW – filtered water (control); H-ERW – hydrogen-enriched electrochemically reduced water; ORP – oxido-reductive potential; DH – dissolved hydrogen; ΔDH/Δt – rate of decay (assumed linear); N/A – not available because of sensitivity limits of the instrument.

# - measured at 37°C;

* - estimated at room temperature in an open glass bottle with minimal agitation.

Dissolved hydrogen has been shown to readily cross all biological membranes and diffuse very quickly in mammalian tissues (see [2]). The elimination rate and routes are not thoroughly described (exhaled mainly), nor are established effective tissue levels that would exert protection. However, concentrations as low as 0.5 ppb appear effective *in vivo* ([62], see also [12]) An important issue when using hydrogen-enriched water is the possible confounding effect of loss of dissolved hydrogen over time, since gaseous hydrogen readily penetrates glass and plastic commonly used in containers [2]. In our experiments, the time it took the dissolved hydrogen in H-ERW to reach control level averaged 8.5 h. Therefore, one can reasonably assume that providing freely moving mice with fresh H-ERW by the beginning of the active period (when the best part of feeding and drinking occurs) would deliver consistent amounts of hydrogen without requiring more frequent change of drinking bottles. For the *in vitro* experiments, DMEM reconstituted with H-ERW contained much less dissolved hydrogen than freshly prepared H-ERW (see Table 3), but the levels were still manifold higher than in control DMEM. Therefore, one can safely assume that molecular hydrogen has been provided in sufficient amounts in cell culture experiments, too, to account for the observed effects.

The animals were randomly assigned to control or molecular hydrogen-enriched electrochemically reduced water (H-ERW) for the entire duration of the experiment (*i.e.* started at least one week before any experimental procedure, and continued until the animal was sacrificed). The water was changed daily in the beginning of the active phase.

Lipopolysaccharide (LPS, serotype O55:B5, Sigma-Aldrich) was dissolved in sterile saline, aliquoted, and stored at −20°C until use. The mice in every cage were randomly assigned to receive either LPS (0.33 mg/kg b.w.; aliquots diluted with sterile saline 30 min prior to administration) or sterile saline (sham treatment) (injected volume 10 ml/kg b.w. in a single i.p. injection). After the injection, the animals were returned to their respective home cages and left undisturbed until the assessment of spontaneous locomotor behaviour.

### Sickness Behaviour Analysis

The animals were weighed daily (10 a.m.) for at least 3 days prior to LPS administration (baseline body weight) and until sacrifice. The sickness behaviour was assessed by recording the spontaneous locomotor activity of mice housed individually in a fresh cage similar in all aspects to the homecage. The mice were videotaped for 15 min after being placed in the new cage, and then moved back to the homecage. All mice in one cage were tested simultaneously. The recordings were analysed offline by overlapping a grid consisting of 12 identical rectangles (3-by-4 array) on the cage floor and counting the number of rectangles the mouse has entered with all four paws during the test period. The exploratory activity of each mouse was recorded in the same conditions at baseline (22 h prior to the LPS challenge), and 2 h after systemic LPS administration. The spontaneous locomotor activity following the LPS challenge was expressed as percentage from baseline activity. The level of suppression of spontaneous locomotor activity was used as exclusion criterion for gene expression analysis. Thus, the mice that did not exhibit a drop of at least 50% in spontaneous locomotion 2 h after LPS injection were not included in further analyses. The data shown here are pooled from all animals killed 4, 24, and 48 h after systemic administration of LPS (sham-control: N = 15; sham-H-ERW: N = 15; LPS-controls: N = 13; LPS-H-ERW: N = 14).

### Spontaneous Locomotor Activity in the Homecage

The spontaneous locomotor activity in the homecage was recorded in the TraffiCage® system (NewBehavior, Switzerland), as described elsewhere [64]. Briefly, the system consists of an array of five radio frequency antennas embedded in a plastic board placed under the cage which detect the presence of implanted radio frequency transponders with a temporal resolution of 20 ms, and it can monitor simultaneously up to 5 mice housed together. Locomotor activity is recorded when one transponder’s detection changes location from one antenna to an adjacent antenna (“crossing”). The time span in which one transponder is detected continuously by one single antenna (“visit”) is used for resting time analysis as follows: a visit (uninterrupted detection by one single antenna) longer than 10 min is considered “resting time”. The spontaneous locomotor activity was calculated as the total number of visits per time interval (hour or phase of the light-dark cycle). Similarly, the total duration of resting time was pooled per time interval (hour or phase of the light-dark cycle). The mice were allowed to acclimate to the system for 2 days before the experiment. Baseline activity was recorded over 2 dark-light cycles prior to treatments, and for 4 dark-light cycles following the LPS injection. The data were analysed using custom-made routines implemented under Matlab R2010b.

### Collection of Brain Material

The animals were killed 2, 4, 24 or 48 h after the injection of LPS. The mice were injected with a lethal dose of pentobarbital and then exsanguinated by transcardial perfusion with ice-cold PBS. The brain was quickly dissected free on ice and cut in half on the midline. One half was fixed by immersion in 14% picric acid in 4% paraformaldehyde overnight at +4°C. The tissue was then cryoprotected in 10% buffered sucrose and stored at −80°C until use. The other half was dissected into several regions and stored at −80°C until use.

### Gene Expression Analysis

The hippocampi were selected for analysis because it has been shown that this particular brain region displays the most robust induction of cytokine expression upon systemic inflammatory challenge by LPS [65], [66] We extracted total RNA using the TRI® reagent (Sigma) according to the manufacturer’s protocol. Briefly, the frozen tissue was thawed and mechanically dissociated in 1 ml TRI reagent. After homogenisation, chloroform was added for phase separation. The RNA was precipitated from the aqueous solution with 2-propanol, and the precipitate was dissolved in 30 µl DEPC water after 2 washes in 75% ethanol. After reverse-transcription, the cDNA was subject to quantitative real time PCR. We analysed the expression of cytokines (proinflammatory: TNF-α, IL-1β, IL-6; anti-inflammatory: IL-10), antioxidant system-related enzymes (Nrf2, HO-1, Cat and iNOS), and BDNF. The PCR reaction contained 1 µl cDNA, 0.2 mM of each primer, and PCR MasterMix (Applied BioSystems, Inc.). The sequences of the primers are listed in Table 4. Product specificity was determined based on melting curve analysis (temperature ramp from 60°C to 95°C) and agarose gel electrophoresis. All amplification reactions were repeated 3 times in independent experiments (N = 4–5 samples/group and timepoint). The expression levels were normalized to the housekeeping gene HPRT1, which was not regulated by LPS administration (ΔCt = Ct_target_−Ct_housekeeping_). The relative expression levels were calculated as ΔΔCt = ΔCt_H-ERW_−ΔCt_control_ (the effect of molecular hydrogen at baseline), or ΔΔCt = ΔCt_LPS_−ΔCt_sham_ (the effect of LPS in controls and H-ERW-treated mice respectively). The changes in expression were calculated as 2^−ΔΔCt^ (fold change) and plotted on logarithmic axes.

**Table 4:** Primers used for rtPCR.

Protein (abbreviation)	Primer sequence
Tumour necrosis factor (TNF-α) [67]	Fwd CATCTTCTCAAAATTCGAGTGACAA
	Rev TGGGAGTAGACAAGGTACAACCC
Interleukin-1β (IL-1β) [67]	Fwd CAACCAACAAGTGATATTCTCCATG
	Rev GATCCACACTCTCCAGCTGCA
Interleukin-6 (IL-6) [67]	Fwd GAGGATACCACTCCCAACAGACC
	Rev AAGTGCATCATCGTTGTTCATACA
Interleukin-10 (IL-10) [67]	Fwd GGTTGCCAAGCCTTATCGGA
	Rev ACCTGCTCCACTGCCTTGCT
Inducible Nitric Oxid Synthase (iNOS)	Fwd CACCTTGGAGTTCACCCAGT
	Rev ACCACTCGTACTTGGGATGC
Nuclear factor-erythroid 2-related factor (Nrf2) [68]	Fwd AGC AGG ACA TGG AGC AAG TT
	Rev: TTC TTT TTC CAG CGA GGA GA
Catalase (Cat)	Fwd ACATGGTCTGGGACTTCTGG
	Rev CAAGTTTTTGATGCCCTGGT
Haeme oxygenase 1 (HO-1) [69]	Fwd GCC TGC TAG CCT GGT GCA AG
	Rev AGC GGT GTC TGG GAT GAG CT
Brain-derived Neurotrophic Factor (BDNF) [70]	Fwd GTGTGTGTCTCTGCGCCTCAGTGGA
	Rev GAAGTGTACAAGTCCGCGTCCTTA
Hypoxanthine-guanine phosphoribosyl-transferase 1	Fwd TGATCAGTCAACGGGGGACA
(HPRT1)	Rev TTCGAGAGGTCCTTTTCACCA

### Cell Cultures

We used the BV-2 cell line (immortalized murine microglia [53], [54]) for testing the effect or H-ERW pretreatment on microglial activation. The cells were seeded at a density of 5000 cells/cm^2^ and grown in DMEM medium supplemented with 10% fetal calf serum (FCS). After 24 h, the medium was changed for serum-free reconstituted DMEM 24 h prior to the exposure to LPS. The cell culture medium was prepared from powder dissolved in either control water or H-ERW according to the manufacturer’s instructions, to yield control-DMEM and H-ERW-DMEM, respectively. The cells were then exposed to LPS (serotype O55:B5, Sigma-Aldrich) (1 µg/ml) for 24 or 2 h (see below).

In order to monitor the proliferation and the state of activation of BV-2 cells, we used an automated cell culture and monitoring system comprising both incubator and imaging hardware (Cell-IQ® 2, Chip-Man Technologies Limited, Tampere, Finland). The BV-2 cells cultures were prepared as described above. The plates were then placed in the Cell-IQ® system immediately after changing the medium to CONTROL- or H-ERW-DMEM. Phase-contrast images were acquired in 3 random locations in each well every 30 min for 24 h. The plates were removed briefly to add LPS to the culture medium, then replaced in the Cell-IQ® system and monitored for an additional 24 h. The experiment was repeated 2 times, with 3 and 6 replicates/experiment. The cells were identified and classified using an automated segmentation algorithm based on a training set of images selected from the acquired stack by the user. For analysis we used the total cell count and the proportion of cells with morphological appearance of activated microglia (*i.e.* large cell body with short and thick processes, in contrast to resting microglia which display a small and slender cell body with few long and very thin processes).

The proliferation of BV-2 cells was assessed for 24 h after changing the culture medium to serum-free, reconstituted DMEM (control- or H-ERW-DMEM, respectively) by fitting the cell count/frame with an exponential curve (*N = N_0_e^kt^*, where *N_0_* is the number of cells counted in the first image acquired for the frame, and *k* is a time constant describing the rate of change in number of cells per time unit). The number of cells/frame varied constantly because of cell divisions and migrating cells moving in and out of the field of view. However, assuming the migration of cells to occur stochastically, the effect on the long-term trend can be ignored; therefore, the long-term trend in cell number is assumed to be due to the balance between cell division and cell death.

The activation of BV-2 cells was estimated by the ratio between the number of cells displaying morphological features of activated microglia and the total number of cells per frame and timepoint. The values were normalized to the average over the last 2 hours before exposure to LPS. The normalized values were averaged cross-sectionally per frame and timepoint for the dynamic phase immediately after the addition of LPS to the culture medium. For later timepoints (12 and 24 h after LPS), the very slow trend allowed averaging the values over 2 consecutive hours centered on the timepoint.

For immunocytochemistry, the cells were harvested 2 h after exposure to LPS and fixed for 1 h in 4% paraformaldehyde, then washed in PBS and processed for staining against microglial activation markers (CD-11b). For gene expression analysis, total RNA was isolated from cells using a PEQLab Gold Total RNA kit according to the manufacturer’s instructions. The isolated RNA was reverse-transcribed into cDNA, which was later subject to quantitative real time PCR analysis using the procedure described above.

### Statistical Analysis

The behavioural data were analysed by factorial ANOVA (factors: water: control/H-ERW; and treatment: sham/LPS). Where available, longitudinal data were analysed by repeated measures ANOVA (long term monitoring of behaviour, body weight), followed by contrast analysis. The relative level of spontaneous locomotor activity and resting time in the homecage after LPS administration was first analysed by one-sample t-test versus the mean 100% to identify the timepoints where there were significant deviations from baseline. The longitudinal between-group comparison was tested using a repeated measurements ANOVA model followed by contrast analysis for individual timepoints (*i.e.* one-tailed *a priori* hypothesis based planned comparisons). Similarly, the gene expression data were analysed first by one-sample hypothesis testing (one sample t-test) to identify the genes and timepoints where significant gene regulation occurred. Where significant regulation was found in at least one of the LPS-treated groups, the data were further analysed using factorial ANOVA (factor 1: treatment; factor 2: timepoint) followed by contrast analysis (*in vivo*) or by student’s t-test (*in vitro*). A one-tailed p-value of 0.05 was set as threshold for significance in all tests. The data are reported as average ± standard error of the mean.

## Supporting Information

Figure S1 **Changes in amplitude of the 3 main peaks of spontaneous locomotor activity displayed during the dark phase.** Repeated measures ANOVA followed by contrast analysis: * p<0.05; # p = 0.06; arrowheads indicate timepoints where only one group was significantly different from baseline.
(EPS)

Figure S2 **Comparison between the expression profile** ***in vivo*** **and** ***in vitro*** **.** This points towards microglia being the main source of alterations in mRNA expression for the proteins investigated during neuroinflammation. However, the interpretation of this observation is limited by a number of factors related to the origin of the samples and the *in vivo vs. in vitro* experimental conditions.* - significant difference between LPS-H-ERW and LPS-control; arrowheads indicate timepoints where the expression was significantly regulated in only one group [see also Tables 1 (4 h) and 2 (2 h)].
(EPS)

File S1 (DOCX)
