# Supplementary material for: Molecular Hydrogen Reduces LPS-Induced Neuroinflammation and Promotes Recovery from Sickness Behaviour in Mice
Source: PLoS One. 2012 Jul 31;7(7):e42078. doi: 10.1371/journal.pone.0042078 (PMC3409143; doi:10.1371/journal.pone.0042078)
Supplement: File S1 — (DOCX) [file pone.0042078.s003.docx]

***Sickness syndrome and the recovery of ultradian patterns of activity***

Both the number and the location (in time) of the peaks of activity during the dark phase are significant. In our hands (group-housed animals monitored continuously on the TraffiCage system), the mice have three distinct peaks of activity with different biological correlates. The locomotor activity starts to increase shortly before turning the light off (about 30 min), then increases abruptly after turning the light off, similar to an awakening reaction. The first peak also coincides with the peak in corticosterone secretion and lasts about 3h. The second peak is located immediately after the middle of the dark period, and coincides with the first consistent bout of feeding and drinking. The fact that the middle peak is clearly identifiable in LPS-H-ERW mice is consistent with the faster recovery in body weight as compared to LPS-controls. The third peak can be interpreted as anticipatory behaviour, because it is very consistently initiated between 1.5 and 1h before ahead of the change of phase and terminates abruptly as the light turns on. Therefore, changes in any of the three peaks are suggestive for abnormal circadian and/or ultradian pattern of spontaneous locomotor activity.

To investigate the alterations in the pattern of activity during the dark phase we have analyzed the activity data in 5min long bins, which would allow a fine localization of amplitude peaks of spontaneous locomotor activity. We then identified and averaged (per animal) the amplitude of the largest peaks located in one of the following time intervals: [9 15); [15 21); [21 +2] (note that the origin of the time axis is set to 0 at the moment of onset of the light phase (zeitgeber (ZG) time), therefore the onset of the dark phase occurs at ZG 12, and the non-overlapping intervals provided here span 6h and are centered on the beginning, the middle, and the end of the dark phase). We then estimated the relative change in amplitude as compared to the baseline (the average of the last 2 24h intervals before the administration of LPS). Importantly, it must be pointed out that the location of the peak of activity does not have any influence on the amplitude, since we did not average over time (as displayed in the figure included in the manuscript), but across individually identified peaks. We found that (Fig S1) (1) the first and the second peaks of activity were suppressed to a lesser extent; and (2) the third peak, although suppressed to a similar extent in both groups, recovers significantly earlier in the LPS-H-ERW group than in LPS-control. Note also that the first and the third peak do not reach the baseline level before the third day after LPS in LPS-control mice, as compared to LPS-H-ERW mice which recover to baseline level within 24h after LPS. We can therefore conclude that the circadian and ultradian patterns of spontaneous locomotor activity recover earlier in the LPS-H-ERW mice than in LPS-controls.
